# Demand for long acting and permanent contraceptive methods and associated factors among married women of reproductive age group in Debre Markos Town, North West Ethiopia

**DOI:** 10.1186/1472-6874-14-46

**Published:** 2014-03-13

**Authors:** Gizachew Abdissa Bulto, Tatek Abate Zewdie, Teresa Kisi Beyen

**Affiliations:** 1 Department of Nursing and Midwifery, College of medicine and health science, Ambo University, Ambo, Ethiopia; 2 Department of Midwifery, College Of Medicine and Health Science, University of Gondar, Gondar, Ethiopia; 3 Department of Epidemiology and Biostatistics, Institute of Public Health, College of Medicine and Health Science, University of Gondar, Gondar, Ethiopia

## Abstract

**Background:**

Ethiopia is the second most populous country in sub Saharan Africa with high total fertility rate, and high maternal and child mortality rates. In sub Saharan African countries, including Ethiopia, even though studies show that demand for contraception is high, the practice is low. Particularly, in Ethiopia, despite the fact that practices on long acting and permanent methods are believed to be low, there are limited evidences on the real magnitude of demand for the methods.

**Methods:**

To assess demand for long acting and permanent contraceptive methods and associated factors among married women of reproductive age group in Debre Markos town, Amhara Regional State, North West Ethiopia, A community based cross sectional study was conducted, from April 08–19, 2012. Systematic sampling technique was used to select 523 study participants. Pre tested structured Amharic version questionnaire was used to collect the data through interview. Both bivariate and multiple logistic regressions were used to identify associated factors.

**Results:**

Among 519 respondents, 323 (62.2%) were using modern family planning (FP) methods in which 101 (19.5%) were using long acting and permanent contraceptive methods (LAPMs). Among all respondents, 171 (32.9%) had unmet need for LAPMs. The total demand for LAPMs was 272 (52.4%) of which 37.1% were satisfied and 62.9% unsatisfied demand. Being in the older age group (40-44 years) [AOR = 2.8; 95% CI:1.12, 9.55], having no desire for more child [AOR = 20.37; 95% CI:9.28, 44.72], desire to have a child after 2 years [AOR = 6.4; 95%CI:3.04,13.47], not ever heard of modern FP [AOR = 5.73; 95% CI:1.26, 25.91], not ever using of modern FP [AOR = 1.89; 95% CI:1.01, 3.55] and having no spousal discussion in the last six month [AOR = 1.642, 95% CI: 1.049, 2.57) were some of the factors significantly associated with demand for LAPMs.

**Conclusions:**

Demand and unmet need for LAPMs were high in the study area. Therefore raising awareness of the community, counseling/discussion about the methods with all clients, encouraging spousal involvement are fundamental areas of intervention. Moreover, increasing the availability and accessibility of LAPMs is required to meet the unmet needs.

## Background

As of July, 2012 world population is estimated to be above 7 billion. Africa accounts around 1 billion people, 15% of the world’s population. The total fertility rates (TFR) worldwide ranges from 1.1 children per women in Taiwan to 7.1 in Niger [1].

Ethiopia is the second most populous country in sub-Saharan Africa (SSA), with an estimated population of over 82 million people, with an average growth rate of 2.6% per year. The population is projected to increase to 166 million by 2050 which makes Ethiopia the 10^th^ most populous country in the world [1,2]. Ethiopia has a TFR of 4.8; ranging from lowest 1.4 children per woman in Addis Ababa to highest 6.2 in Oromia [3]. Studies in countries where fertility is high have showed that, maternal, infant and child mortality rates are high [4,5]. In Ethiopia there are high maternal mortality ratio (676/100,000 live births), high abortion rate (23/1000), and unwanted pregnancy rate (42%) [2,3,6].

The SSA region’s unmet need for family planning (FP) is the highest in the world (48.8 million women), nearly half the married women of reproductive age (MWRA) want to space or limit the number of children they have. However, less than one in seven MWRA (14.7 million) are using a modern method of contraception, of which only 2.7 million MWRA use long-acting or permanent contraception [7,8]. Reasons for unmet need are lack of knowledge about contraceptive options and their use, unavailability of services, limited supplies everywhere or choices, fear of social disapproval or partners’ opposition, fear of side effects and health concerns [9].

The 2011 Ethiopian demographic and health survey (EDHS) showed that 38% of women wants child after two years and 37% reported they want no more children. Only 25% of currently married women have an unmet need for FP (16% for spacing and 9% limiting). Total unmet need is highest in Oromia (30%) and lowest in Addis Ababa (11%). Only 29 percent of currently married women are using a method of FP; despite the government is working toward a contraceptive prevalence rate (CPR) of 60% by 2010. Prevalence of LAPMs use constitutes 3.4%, 0.3% and 0.5% for Implant, IUD and sterilized respectively [3,10].

Better availability of FP services, including long acting & reversible methods (LARMs) would fulfill the need for healthier timing and spacing of pregnancies [11]. Four contraceptive methods are categorized under LAPMs: intrauterine devices (IUDs), implants (Implanon, Jadele), female sterilization, and vasectomy. IUDs and implants are long-acting temporary methods; when removed, return to fertility is prompt; and female sterilization and vasectomy are permanent method. Implants are effective from 3 to 7 years depending on type and IUDs are effective for at least 12 years [12].

LAPMs are the most effective (99% or greater) methods of contraception available and are very safe and convenient for protection against unintended pregnancy [9]. Experience in countries where LAPMs are available shows; they are highly popular than short acting methods [12]. According to world health organization (WHO) eligibility criteria, almost all women are eligible for IUDs, implants and sterilization [13]. They are also cost effective for programs over time and with lowest discontinuation rates. Despite these advantages, LAPMs remain a relatively small and sometimes missing component of many national reproductive health and FP programs [8,11,12]. Global experience including the SSA countries confirmed that without widespread availability and use of LAPMs of contraception, a country cannot meet its lowering fertility goals, reducing maternal and child mortality. Investing in FP specifically on LAPMs is one of the key for reducing child and maternal mortality; and to halt/combat HIV/AIDS directly and to achieve other MDG goals indirectly [11,12].

In Ethiopia despite the fact that the practices on long acting and permanent methods are believed to be low, there are limited recently available evidences on the real magnitude and associated factors of the demand for LAPM. Thus, there is a need to assess the demand for LAPMs and associated factors among married women of reproductive age in the study area.

## Methods

### Study design and area

To assess demand for long acting and permanent contraceptive methods and associated factors among married women of reproductive age group in Debre Markos town, East Gojjam zone, Amhara national regional state (ANRS), North West Ethiopia, a community based cross sectional study was conducted from April 08–19, 2012. Debre Markos town is located at 300Km Northwest of Addis Ababa and 265Km southeast of the Regional capital city, Bahir Dar. The town is divided in to seven kebeles (small administrative regions) [14]. Based on the 2007 population and housing census, the total population size of the town is estimated to be 62,469 of which 47.9% were male and 52.1% were females [2]. Out of the total females, women in the reproductive age group were 14,618 and the numbers of households in the town were estimated to be 14,528. According to information from the town’s health office the health system of the town consisted of one Referral Hospital, three Health center, and two none governmental organizations (NGOs) clinics (family guidance association clinic (FGA) and Marie stops international clinics) providing reproductive health services including LAPMs. All married women in the reproductive age group (15- 49 years) who lived in the Debre Markos town and fecund were considered for the study. However, married women in the reproductive age who were seriously ill and unable to hear were excluded.

### Sampling procedure and sample size

**Figure 1:**
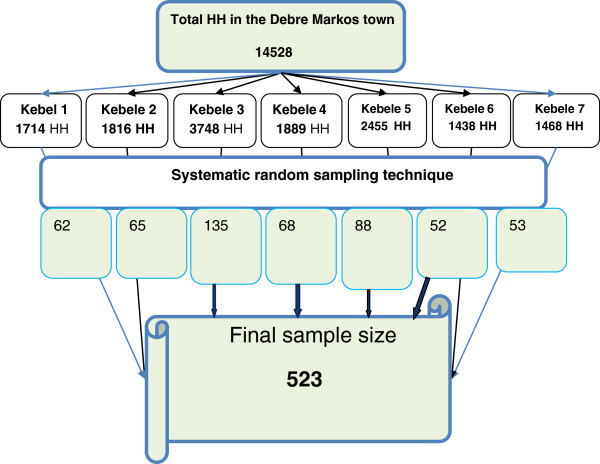
Schematic presentation of sampling procedure of married women of reproductive age in Debre Markos town, North West Ethiopia April 2012.

The sample size was calculated by using single population proportion formula by considering 50% proportion of demand for LAPMs, since we couldn’t found similar study, specifically on demand of LAPM, 95% confidence level, 4.5% Margin of error and 10% none response rate. The final sample size used was 523. Systematic random sampling technique was employed to select the house hold from each kebeles by considering that there was at least one married women per house hold and using the number of house hold as a sampling frame. The first households were selected from the town using the town’s house number registration by lottery method. It has been determined that the households were selected every 28^th^ interval (i.e. by dividing the total households (H) to the sample size (h)) using the first selected household as reference. In cases of selected household with more than one eligible respondent, only one respondent was chosen by lottery method. In cases where no eligible participant identified in the selected household, the data collectors have gone to the next household to the right direction until they got eligible women (Figure 1).

### Data collection procedures

Data were collected by face to face interview by using structured and pre tested Amharic version questionnaire. The questionnaires were first prepared in English and translated in to Amharic and back to English by language experts to keep consistency of the questionnaires. The questions included in the questionnaire were prepared from different related literatures. Seven well trained diploma nurses graduated from private collages collected data and two BSC midwives have supervised during data collection period. The filled questionnaires were checked for consistencies and completeness daily by supervisors and principal investigators on the spot. Pre -test of the questionnaire were done on 5% of the sample on women of reproductive age group nearby Debre Merkos town (i.e. at Finote selam town), to identify any ambiguity, consistency and acceptability of questionnaire, and then necessary corrections were made before the actual data collection.

### Data analysis and measurement procedures

The collected data were cleaned, coded, and entered in to EPI info 2011 and then exported to SPSS version 20 for further analysis. A women had unmet need for reversible long acting methods when she have desired to delay or avoid pregnancy for 2 years or more but not used implants or IUD, or those who were using unsuitable short acting methods. Unmet need for permanent methods was considered when a women who have achieved desired family size (don’t want any more), but were not using LAPMs or unsuitable other methods. Then demand for LAPMs was defined as the sum of those women who were using LAPMs and had both unmet need for reversible long acting and permanent methods. Both descriptive and analytical statistics were used for data analysis and interpretations. Bivariate analysis was conducted primarily to check the variables which had an association with the dependent variable individually. Variables associated with the dependent variables at p value 0.2 were then entered in to multiple logistic regression for controlling the possible effect of confounders and finally the variables which had significant association with demand for LAPM were identified on the basis of OR, with 95% CI and p-value (<0.05) to fit into the final regression model.

### Ethical considerations

Before actual data collection, ethical clearance was obtained from Institute of public health research ethical review committee of University of Gondar. Formal letter of cooperation was written to Debre Markos town health office and it was also obtained from Debre Markos town health office to each Kebele administrations. Informed consents were obtained from each study participants after the objectives of the study was fully explained in their local languages. Participants who refused to participate in the study were not forced and the collected data were stored in a file, without the name of study participant (anonymously), but code was assigned for each and had not been disclosed to others except to the principal investigators.

## Results

### Socio demographic characteristics of respondents

A total of 519 married women in the reproductive age were responded for the study making the response rate of 99.24%. The mean age of respondents was 29.64 years with 7.65 years Standard deviations. Majority of the study subjects were Amhara in ethnicity (93.3%) and 422 (81.3%) of them were Orthodox followers. Concerning educational status, 156 (30.1%) of respondents were secondary school and 101 (19.5%) of them were unable to read and write. On the other hand, majority (37.4%) of respondents’ husband had educational status of above 12 grades, followed by secondary education (25.4%). Two hundred thirty five (45.3%) of respondents were housewife and 219 (42.2%) of their husbands were governmental employee (Table 1).

**Table 1:** Socio-demographic characteristics of married women in the reproductive age group in Debre Markos town, North West Ethiopia, April 2012 (n = 519)

**Variables**		**n (%)**
**Age**	15–19	30 (5.8)
	20–24	120 (23.1)
	25–29	129 (24.9)
	30–34	85 (16.4)
	35–39	84 (16.2)
	40–44	52 (10.0)
	45–49	19 (3.7)
**Ethnicity**	Amhara	484 (93.3)
	Oromo	12 (2.3)
	Tigre	10 (1.9)
	Agawu	10 (1.9)
	Others*	3 (0.6)
**Religion**	Orthodox	422 (81.3)
	Muslim	67 (12.9)
	Protestant	20 (3.9)
	Catholic	10 (1.9)
**Respondents educational status**	Can’t read & write	101 (19.5)
	Read and write only	71 (13.7)
	Primary school (1–8)	95 (18.3)
	Secondary (9–12)	156 (30.1)
	Above 12	96 (18.5)
**Husbands education status**	Can’t read & write	36 (6.9)
	Read and write only	74 (14.3)
	Primary school (1–8)	83 (16.0)
	Secondary (9–12)	132 (25.4)
	Above 12 grade	194 (37.4)
**Respondents occupational status**	House wife	235 (45.3)
	Merchant	101 (19.5)
	Farmer	12 (2.3)
	Daily labourer	50 (9.6)
	Government employee	100 (19.3)
	Student	21 (4.0)
**Husband’s occupation**	Merchant	139 (26.8)
	Farmer	45 (8.7)
	Daily labourer	95 (18.3)
	Government employee	219 (42.2)
	Private work	21 (4.0)

*Gurage, wolayita.

### Reproductive history of the respondents

**Figure 2:**
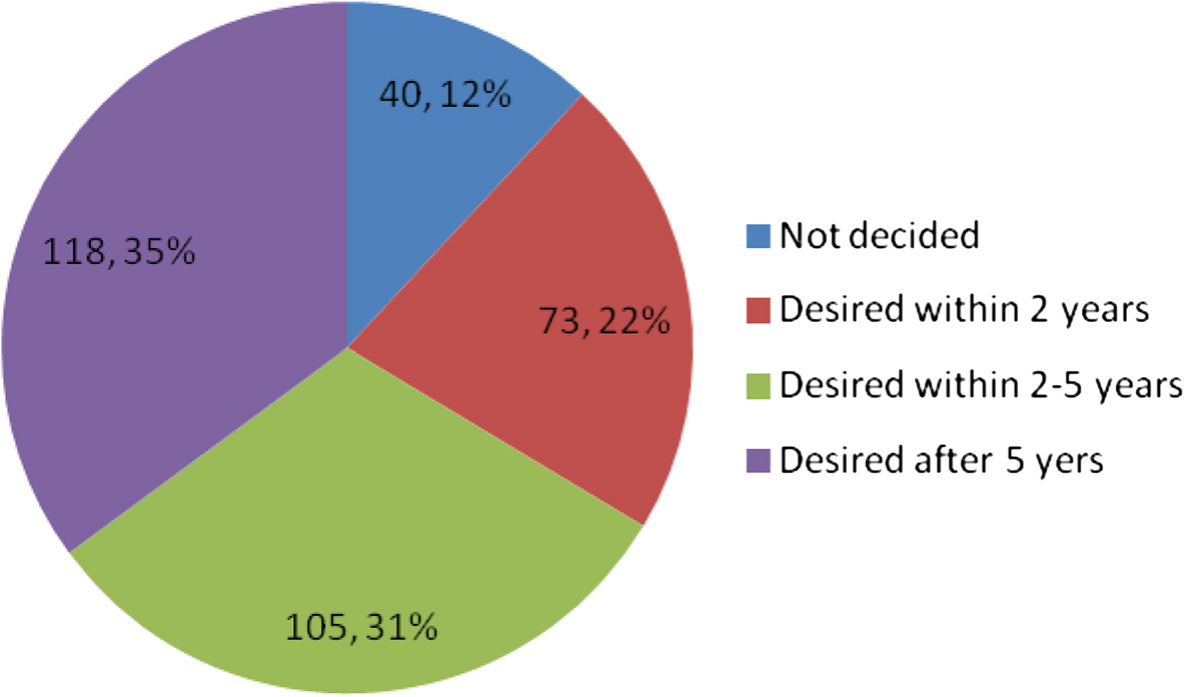
Fertility intention of married women of reproductive age group in Debre Markos town, North West Ethiopia, April 2012.

Out of the total respondents, 385 (74.2%) have given birth to one or more children. Forty six (8.9%) of respondents were pregnant during the study period. Out of these, 30 (65.2%) were desired pregnancies, 11 (23.9%) pregnancies were mistimed and 5 (10.9%) were not wanted at all. Three hundred thirty six (64.7%) of the study participants desired to have a child and 183 (35.3%) of them didn’t desire to have a child in the future which are considered here after as those with the intention to limit child bearing (Table 2). Among those who desired to have a child in the future 118 (35%) of them wanted within 2–5 years and 105 (31%) wanted after 5 years (Figure 2). The average numbers of children desired in life, ever born and living child were 3.32 with SD of 1.06, 1.97 with SD of 1.67 and 1.84 with SD of 1.56 respectively.

**Table 2:** Reproductive history of married women in the reproductive age in Debre Markos town, Northwest Ethiopia, April 2012 (n = 519)

**Variables**		**n (%)**
**Ever given birth**	Yes	385 (74.2)
	No	134 (25.8)
**Current pregnancy status**	Pregnant	46 (8.9)
	Not pregnant	473 (91.1)
**Desire for more child**	Yes	336 (64.7)
	No	183 (35.3)
**Number of desired children**	1	3 (0.6)
	2	146 (28.1)
	3	135 (26.0)
	4	154 (29.7)
	5+	81 (15.6)
**Number of children ever born**	None	134 (25.8)
	1	95 (18.3)
	2	110 (21.2)
	3	78 (15.0)
	4	36 (6.9)
	5+	66 (12.7)
**Number of children alive**	None	136 (26.2)
	1	102 (19.7)
	2	118 (22.7)
	3	79 (15.2)
	4	41 (7.9)
	5+	43 (8.3)

### Knowledge/awareness of women about long acting and permanent methods and source of information

**Figure 3:**
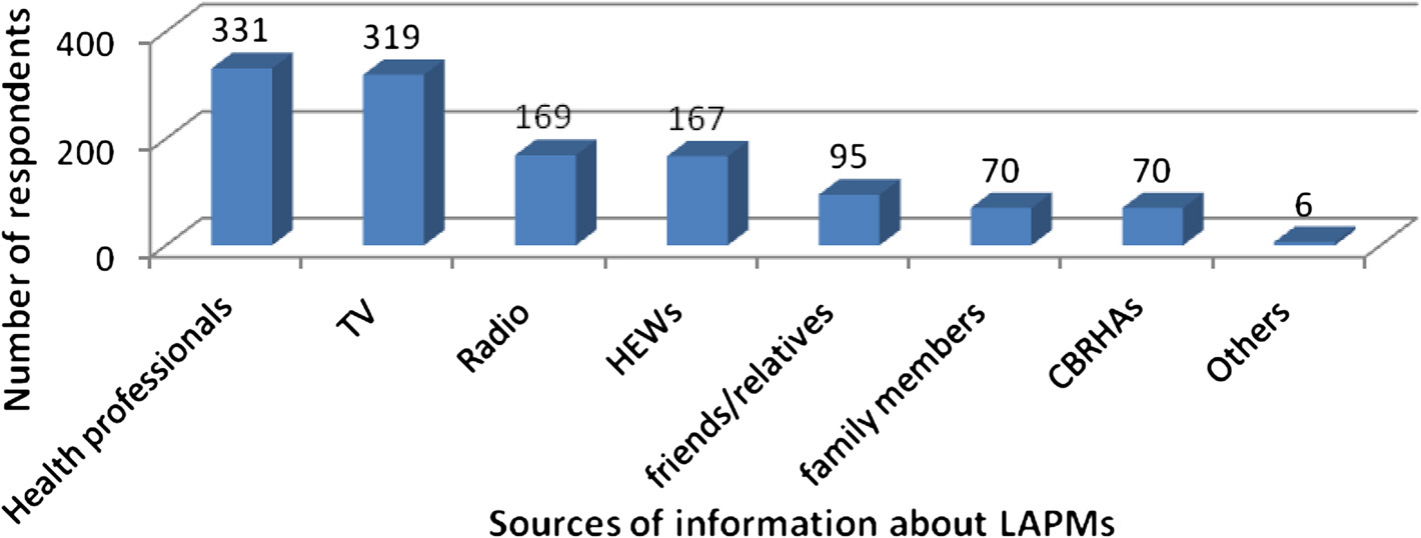
Source of information about LAPM contraceptive methods among married women of reproductive age in Debre Markos town, North West Ethiopia, April 2012.

**Figure 4:**
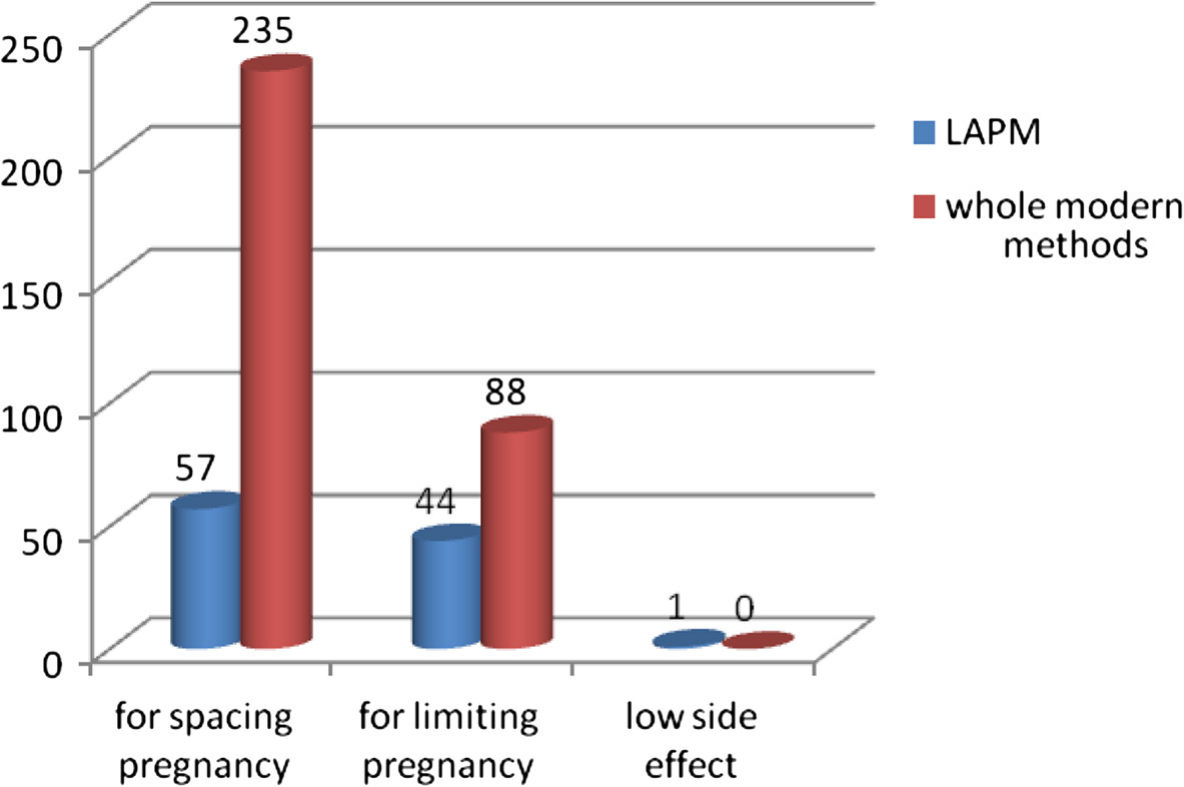
Purpose of use of modern contraceptives and LAPMs mentioned by married women of reproductive age in Debre Markos town, North West Ethiopia April 2012.

Five hundred two (96.7%) of the respondents have heard at least one methods of modern Family planning. Among the methods, Injectable was mentioned by 501 (96.5%) respondents followed by daily pills (80.3%). Four hundred twenty three (81.5%) of respondents knew/mentioned at least one methods of LAPMs contraception and 96 (18.5%) didn’t know any LAPMs methods. Health care professionals (63.8%) and television (61.5%) were the most commonly mentioned source of information (Figure 3). About 415 (80%) respondents knew at least one place where LAPMs could be obtained. Health center (74.6%), hospital (58.0%), NGO clinics (12.7%) and health posts (12.3%) were the commonly mentioned sites where LAPMS could be obtained. Spacing births (56%) and limiting family size (43%) were the commonly mentioned advantages of LAPMs (Figure 4). Two hundred seventy three (52.6%) of respondents had discussion about LAPMs at least once with the health care providers and the most commonly discussed methods were Implanon (45.5%) where as male sterilization (3.1%) were the least mentioned (Table 3).

**Table 3:** Knowledge and discussion with health professionals about LAPMs among married women in the reproductive age in Debre Markos town; North West Ethiopia, April 2012 (n = 519)

**Variables**		**n (%)**
Contraceptive methods known by women	Injectable	501 (96.5)
	Daily pill	417 (80.3)
	Jadele	384 (74.0)
	Implanon	367 (70.7)
	IUD	294 (56.6)
	Female sterilization	133 (25.6)
	Condom	105 (20.2)
	Male sterilization	56 (10.8)
	Emergency pills	53 (10.2)
Knowledge of advantage of LAPMs	Spacing of births	384 (74.0)
	Limiting family size	332 (64.0)
	Highly effective method	76 (14.6)
	Have lower side effects	38 (7.3)
	Reduce cost	8 (1.5)
LAPMs ever discussed with health professionals	Not discussed	246 (47.4)
	Implanon	236 (45.5)
	Jadele	198 (38.2)
	IUD	120 (23.1)
	Female sterilization	63 (12.1)
	Male sterilization/vasectomy	16 (3.1)
Frequency of discussion in the last one year	none	246 (47.4)
	Once	112 (21.6)
	Twice	80 (15.4)
	Three times	33 (6.4)
	Four and above	48 (9.2)

### Modern contraceptive practices among married women of reproductive age

**Figure 5:**
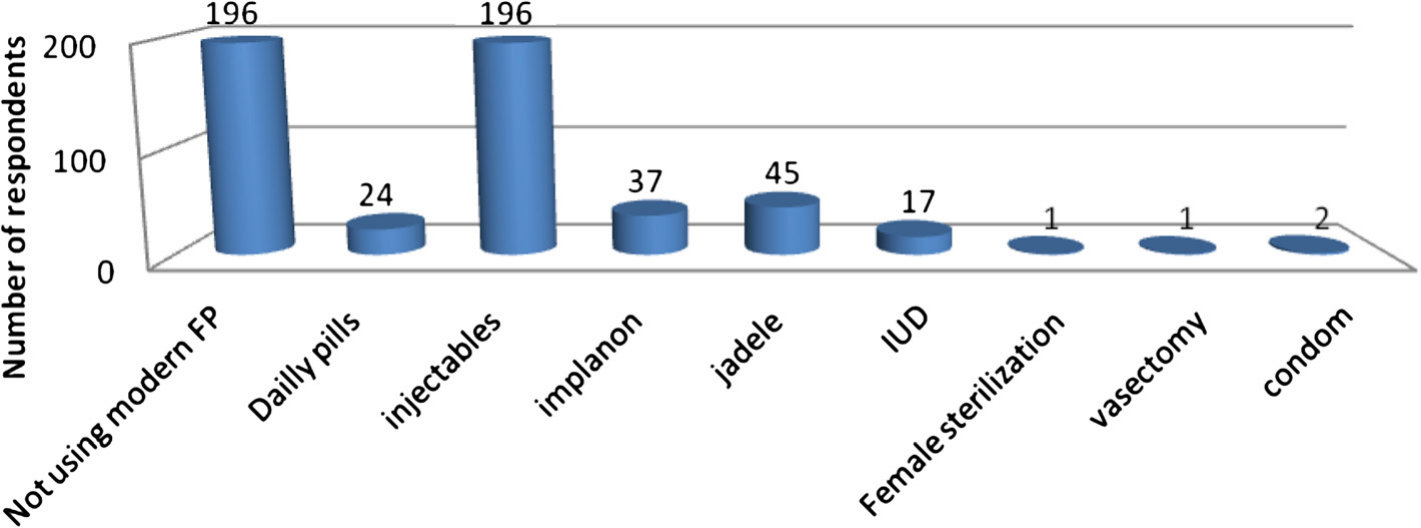
Modern contraceptive methods practiced among married women of reproductive age in Debre Markos town North West Ethiopia, April 2012.

Out of total respondents, 406 (78.2%) have ever used modern contraceptive methods and the mean duration of use were 40.9 months with SD of 25.8 months. Three hundred twenty three (62.2%) respondents were using modern family planning methods of which injectables (60.7%) were the most used family planning method (Figure 5).

One hundred one (19.5%) respondents were using LAPMs, of which 37 (36.7%) were using Implanon, 46 (45.5%) were using Jadele and 18 (17.8%) were using IUD. Forty two (76.4%) of users of implants or IUD wanted to continue their method up to the end and 13 (23.6%) of them wanted to remove before the actual date because of their need to be pregnant.

### Respondents’ intention to use LAPMs, discussion with their partners, reasons for using and not using LAPMs

Among total respondents, 371 (71.5%) couples approved using LAPMs and 148 (28.5%) of them didn’t approve. Two hundred thirty eight (45.9%) of women had intention to use one of the LAPMs of contraception in the future. Ninety eight (18.9%) of the respondents intended to use Implanon, 67 (12.9%) Jadele, 60 (11.6%) IUD and 13 (2.5%) female sterilization. The reasons mentioned for intention to use LAPMs were; wanting longer interval between pregnancies or spacing (55.9%) and don’t wanting any more child (28.2%).

Two hundred thirty five (45.3%) of respondents have discussed about contraceptive methods with their husband in the last six months of which, 87 (16.8%) of them have discussed twice. Only 281 (54.1%) respondents’ husband approved using LAPMs and 130 (25.0%) respondents didn’t know their husbands attitude. Three hundred ninety (75.1%) respondents’ husbands knew their status of contraception and 336 (64.7%) have discussed with their spouse about which method to use. About 191 (36.8%) respondents perceived that their husband should approved using of permanent methods after completing desired family size. Majority (70.5%) of respondents believed joint decision about to use LAPMs with their partner (Table 4).

**Table 4:** Intention to use LAPMs and spousal discussion among married women of reproductive age groups in Debre Markos town, North West Ethiopia, April 2012 (n = 519)

**Variables**		**n (%)**
**Intention to use LAPMs in the future**	Wants to use LAPMs	238 (45.9)
	Don’t want to use LAPMs	194 (37.3)
	I don’t know	87 (16.8)
**Reasons for intending to use (N = 238)**	To space for longer interval	133 (55.9)
	Don’t want to have more child	67 (28.2)
	Side effects of others	31 (13.0)
	Not available before	16 (6.7)
	Don’t know its presence	11 (4.6)
**Discussion with partner within last 6 month**	None	284 (54.7)
	Once	71 (13.7)
	Twice	87 (16.8)
	Three times	37 (7.1)
	Four and above	40 (7.7)
**Attitude towards Husbands/approval of using LAPMs**	Approve	281 (54.1)
	Didn’t approve	108 (20.8)
	I don’t know	130 (25.0)
**Husbands knowledge of use or not using of contraceptives**	Yes	390 (75.1)
	No	83 (16.0)
	I don’t know	46 (8.9)
**Spousal discussion about which method to use**	Yes	336 (64.7)
	No	183 (35.3)
**Partner approval of having permanent method after completed family size**	Yes	191 (36.8)
	No	168 (32.4)
	I don’t know	160 (30.8)
**Decision about using LAPMs**	Mainly me	135 (26.0)
	Mainly my husband	18 (3.5)
	Joint decision	366 (70.5)

**Figure 6:**
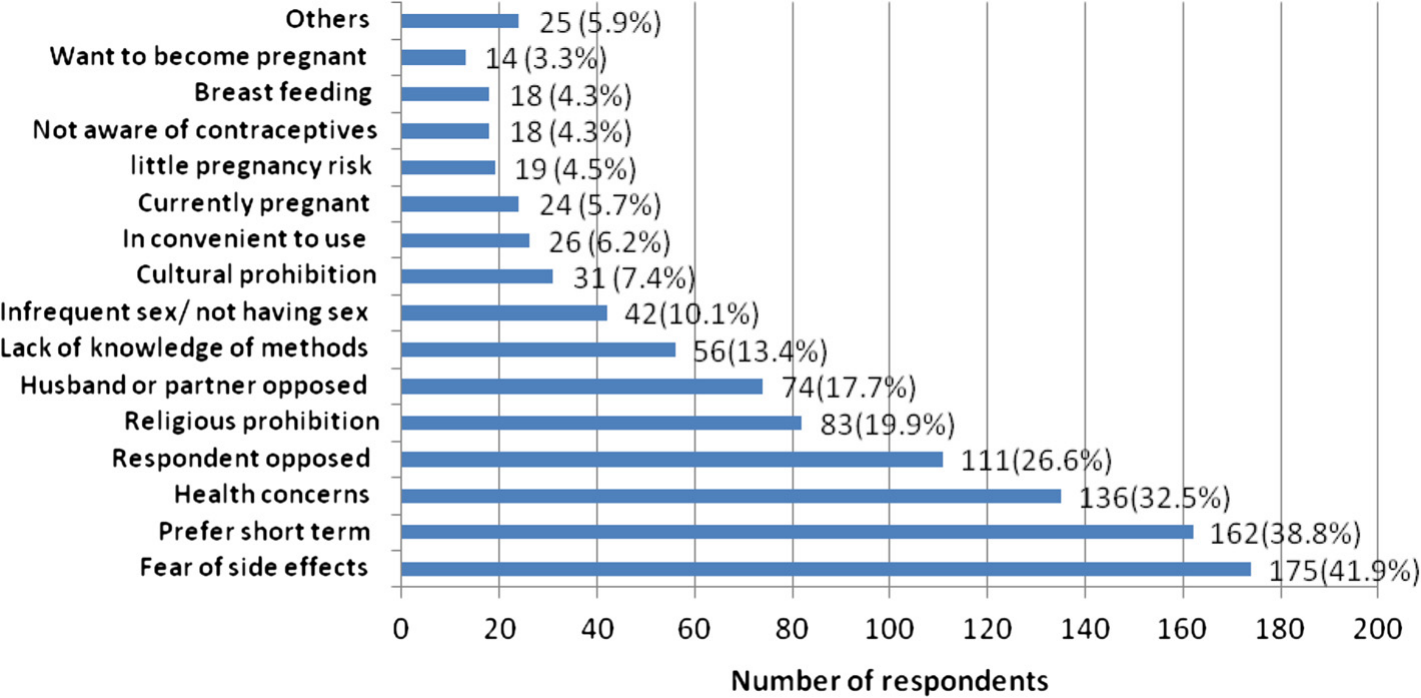
Reasons mentioned for not using LAPMs by married women of reproductive age in Debre Markos town, North West Ethiopia, April 2012.

Out of 418 respondents who are not currently using LAPMs, the main top five reasons mentioned for not using LAPMs were fear of side effects (41.9%), preferring short term (38.8%), health concerns (32.3%), respondents opposed (26.6%) and religious prohibition (19.9%) (Figure 6).

**Figure 7:**
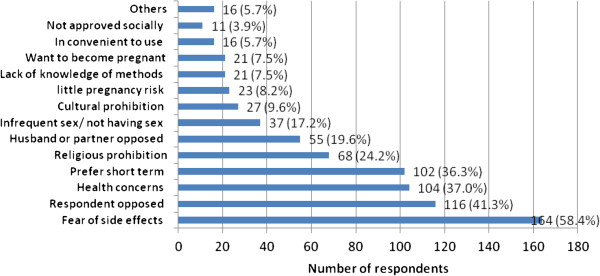
Reasons mentioned for not intending to use LAPMs by married women of reproductive age in Debre Markos town, North West Ethiopia, April 2012.

The top five reasons mentioned for not intending to use LAPMs in the future were; fear of side effects (58.4%), respondents opposed (41.3%), health concerns (37.0%), preferring short term (36.3%) and religious prohibition (24.2%) (Figure 7).

Two hundred forty six (47.4%) of respondents have heard myths/beliefs and traditional misconceptions about at least one methods of LAPMs and majority of them were heard towards implant (31.6%), followed by towards IUD (15.2%), female sterilization (5.0%) and 22 vasectomy (4.2%). The commonly heard myths towards implants were; work related problems (50.0%), menstrual abnormalities (33.5%) and health problems (15.9%). About two fifth and one fifth of the respondents heard that IUD makes infertile and causes genital infections respectively. Ten (38.5%) of respondents said female sterilization makes infertile and twenty (90.9%) of them said vasectomy causes inability to erect penis and makes unable to have sex (Table 5).

**Table 5:** Myths or beliefs and traditional misconceptions heard by married womens in the reproductive age in Debre Markos town, North West Ethiopia, April 2012

**Variables**		**N (%)**
**Heard myths (N = 519)**	**Yes**	246 (47.4)
	**No**	273 (52.6)
**Implants myths (N = 164)**	Prevents from daily work (weakens, tingling & numbness of arm/hand)	82 (50.0)
	It brings menstrual abnormalities	55 (33.5)
	It has many health problems	26 (15.9)
	It makes infertile	20 (12.2)
	It makes irritable or brings behavioral change	20 (12.2)
	Brings hypertension or raises blood pressure	19 (11.6)
	It causes headache and blurring of vision	17 (10.4)
	Weight loss or makes thin	16 (9.8)
	Others*	17 (10.4)
**IUDs myths (N = 79)**	It makes infertile	33 (41.8)
	It brings/causes genital infection	17 (21.5)
	It has many health problems	17 (21.5)
	Causes menstrual irregularity	11 (13.9)
	It may decompose within the womb/uterus	8 (10.1)
	High chance of expulsion & pregnancy may occur	6 (7.6)
	Interferes with sexual activity	5 (6.3)
	Others **	21 (26.6)
**Female sterilizations myths (N = 26)**	Makes infertile	10 (38.5)
	It has many health problems and life threatening	7 (26.9)
	It needs major operation	4 (15.4)
	Predispose to uterine infection	4 (15.4)
	Decreases sexual desire	3 (11.5)
**Vasectomies myths (N = 22)**	Inability to erect or weakens penis/impotency	20 (90.9)
	It has health problems	3 (13.6)

*weight gain, facial change, cause to have twin pregnancy, decrease sexual desire.

**decrease sexual desire, burning genitalia, brings uterine cancer, abdominal distention.

### Demand for long acting and permanent contraceptive methods

**Figure 8:**
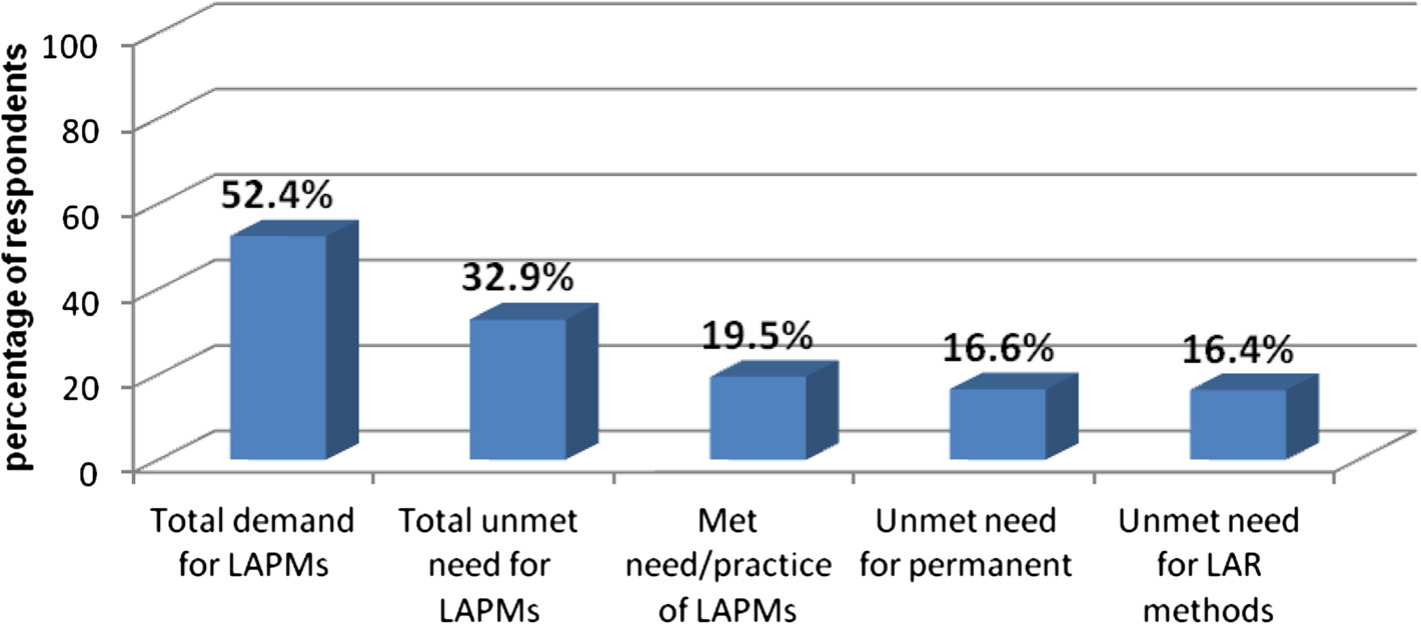
Total Demand, met need and unmet need for LAPMs among married women of reproductive age in Debre Markos town, North West Ethiopia April 2012.

From total respondents, 85 (16.4%) had unmet need for long acting reversible methods and 86 (16.6%) had unmet need for permanent methods. The total demand, which is the sum of those who were practicing LAPMs, 101 (19.5%), and those who had an unmet need for the methods, 171 (32.9%), were 272 (52.4%) [Of which, 37.1% were satisfied and 62.9% were unsatisfied demand] (Figure 8).

### Factors associated with demand for long acting and permanent methods

In the bivariate logistic regression analysis, Demand for LAPMs was associated significantly with age, respondents’ education, husbands’ occupational status, need for pregnancy, current pregnancy status, number of children desired, number of children ever born, number of children alive, knowledge of modern FP methods, knowledge of LAPMs, hearing about LAPMs from health professionals or radios, ever use of modern FP methods, duration of use, current use of contraceptives, ever use of LAPMs before, spousal discussion in the past 6 month about contraception, spousal discussion about contraception, frequency of discussion, husbands’ knowledge about use or non use of contraception, spousal discussion about which methods to use, perception of partner’s approval of using permanent methods after completing family size and decision about to use LAPMs. However, in the multivariate logistic regression analysis, demand for LAPMs was associated significantly with age, desire for more child, duration of desire to have child, number of children ever born, ever heard of modern FP, ever use of modern FP, spousal discussion in the past 6 month about modern family planning, spousal discussion about which method to use and perception of partner’s approval of permanent methods after completing family size (Table 6).

**Table 6:** Factors associated with Demand for LAPMs contraceptives among married women of reproductive age in Debre Markos town, North West Ethiopia, April 2012

**Independent variable**		**Demand for LAPMs**		**Crude OR (95% CI)**	**Adjusted OR (95% CI)**	**P value**
		**Yes**	**No**			
**Age**	15-19	17	13	1	1	
	20-24	49	71	0.528 (0.235-1.185)	0.43 (0.179-1.036)	0.060
	25-24	62	67	0.708 (0.318-1.576)	0.601 (0.253-1.43)	0.250
	30-34	39	46	0.648 (0.28-1.50)	0.625 (0.252-1.545)	0.308
	35-39	50	34	1.125 (0.484-2.614)	1.195 (0.522-3.464)	0.702
	40-44	40	12	2.55 (0.968-6.713)	2.819 (1.129-9.553)	0.049
	45-49	15	4	2.87 (0.767-10.717)	3.33 (0.843-13.176)	0.086
**Desire for more child**	Yes	138	198	1	1	
	No	134	49	3.924 (2.649-5.812)	20.376 (9.28-44.72)	0.000
**Duration of desire for child**	Within two years	144	112	1	1	
	After two years	128	135	0.737 (0.522-1.042)	6.4 (3.04-13.478)	0.000
**Number of children ever born**	None	62	72	1	1	
	**1**	39	56	0.809 (0.475-1.376)	0.792 (0.460-1.366)	0.402
	**2**	56	54	1.204 (0.727-1.996)	1.311 (0.778-2.208)	0.309
	**3**	46	32	1.669 (0.949-2.936)	2.254 (1.218-4.171)	0.010
	**4**	18	18	1.161 (0.556-2.425)	2.34 (1.04-5.26)	0.041
	**5+**	51	15	3.95 (2.02-7.70)	9.0 (3.74-21.74)	0.000
**Ever heard at least one of modern FP methods**	Yes	257	245	1	1	
	No	15	2	0.14 (0.032-0.618)	5.7 (1.27-25.91)	0.023
**Ever use of modern FP**	Yes	184	222	1	1	
	No	88	25	4.25 (2.61-6.90)	1.89 (1.01-3.55)	0.048
**Discussion with partner in the last 6 month**	yes	98	137	1	1	
	No	174	110	2.21 (1.55-3.15)	1.64 (1.05-2.57)	0.030
**Discussion with partner on method to use**	Yes	149	187	1	1	1
	No	123	60	2.573 (1.766-3.748)	2.15 (1.32-3.49)	0.002
**Partner approval of permanent FP method**	Yes	98	93	1.26 (0.82-1.91)	1.93 (1.22-3.07)	0.005
	No	101	67	1.8 (1.159-2.785)	1.64 (1.04-2.59)	0.033
	I don’t know	87	87	1	1	

*AOR*; Adjusted odds ratio, *COR*; Crude odds ratio.

## Discussion

The study revealed that more than half (52.4%) of respondents had demand for LAPMs; of which 32.9% had an unmet need for LAPMs (16.4% for spacing & 16.6% for limiting) and 19.5% were using the methods (11.0% for spacing & 8.5% for limiting). Among the total demand only 37.1% were satisfied where as majority (62.9%) were unsatisfied for the demand of LAPMs in which they required it for spacing or limiting but were not using LAPMs.

The unmet need was lower than study in Congo in which 21% had unmet for spacing and 31% for limiting; and in Rwanda 27% wanted to stop having children of which 57% were not practicing using any contraception or were having an unmet need for permanent methods of FP [15,16]. The reason for this may be due to the differences in time, socio cultural, geographical and study settings. EDHS 2011 showed 25% unmet for FP and total demand of 54% (33% were for spacing and 21% for limiting). From survey in southern nation, nationalities, and peoples’ region (SNNPR) the total demand for FP in the region was 49.3% of which 37.4% had unmet need and 11.9% had met need for FP [3,17]. The reason for this might be the difference in time and study setting in which those studies were conducted in both rural and urban setting and for the general FP methods. However, this study is only for urban and on demand of LAPMs. On the other Hand the current study’s demand, unmet need and practice for LAPMs were higher than studies done in Goba [demand for LAPMs was 18.12% and 9.4% had unmet need] and Batu Jira [total demand for LAPMs among FP service users was 24.4% (3% satisfied and 22.4% unsatisfied)] towns [18,19]. This might be due to the time differences in which currently deferent Medias are promoting LAPMs, so that they may increase the acceptance/practice of LAPMs.

In this study age, desire for more child, duration of desire to have child, number of children ever born, ever heard of modern FP, ever use of modern FP, spousal discussion in the past 6 month about contraception, spousal discussion about which method to use and perception of partner’s approval of permanent methods after completing family size were significantly associated with demand for LAPMs.

Accordingly women whose age were in between 40–44 years were almost three times more likely to have demand for LAPMs than women whose aged were in between15-19 years [AOR = 2.81, CI:1.12-9.55]. This result is supported by study done in Batu jira town [19]. The reason for the age deference could be due to those women who were older were having more children and have more desire to limit or space the number of pregnancy than younger’s who had none or few children. This is also supported by studies done in Goba town, Oromia region, rural Ethiopia and Pakistan, in which women who want more children were younger [18,20-22].

In this study women who had completed their family size or don’t want any more children were twenty point three seven times more likely to have demand for LAPMs than those who desire more [AOR = 20.37; 95%CI: 9.28, 44.72]. This result is supported by study done in Pakistan which revealed that woman who wanted no more child were more likely to intend using contraceptive methods particularly sterilization [22].

Our study revealed that women who wanted to have a child after two years were six point four times more likely to have demand for LAPMs than those who want within two years [AOR = 6.4; 95% CI: 3.04, 13.47]. On the other hand Women who had three, four and five or more ever born child were two point two six, two point two three three, and nine times more likely to have demand for LAPMs when compared to those who had no child [AOR = 2.26, 95% CI: 1.22, 4.17], [AOR = 2.34; 95%CI: 1.04, 5.26] and [AOR = 9.02, CI: 3.74, 21.74] respectively. This result in line with the result of study done in Batu Jira town which showed women with 1–3 and 4–12 children were having fifty one and six times higher demand than who had no child respectively [19]. The reason for deference might be due to the more child the woman is having the more likely she want to space or limit the number of child she have and the more she were using LAPMs or had an unmet need. It is also supported by studies done in Goba, Oromia, Mekelle, Egypt and Uganda [18,20,23-25].

The study revealed that women who have heard of at least one modern FP method were about six times more likely to have demand for LAPMs than those who didn’t [AOR = 5.73; 95% CI: 1.26, 25.91] and women who had ever used modern FP before were almost two times more likely to have demand for LAPMs than those who didn’t [AOR = 1.89, 95% CI: 1.01, 3.55]. Similarly from study in Rwanda demand was higher among women who received information about family planning at health facilities than among those who received no information or those who did not attend such a facility [16]. Also from study done in Goba town and on other Ethiopian districts, family planning practice was significantly associated with willingness to use LAPMs in the future than who didn’t and respondents who had ever used modern family planning were more than seventeen times more likely to use LAPMs than those who didn’t [18,26].

The study indicated that spouse who had no discussion about contraception in the past six months and women who had no discussion about which method to use were one point six and about two times more likely to have demand for LAPMs than those who had discussion [AOR = 1.64; 95% CI: 1.04, 2.57] and [AOR = 2.14; 95% CI: 1.31, 3.49] respectively. On the other hand women who perceived that their husbands would approve when long Acting and permanent methods needed after completing family size were almost two times more likely to have demand for LAPMs than who didn’t know their husbands perception [AOR = 1.93; 95% CI:1.22, 3.07]. This might be due to those who had discussion were more likely to discus on the desired family size, knew their husbands perception towards LAPMs, and desired to use suitable methods than having an unmet need for the methods. The result is supported by studies done in Goba, Pakistan, mekelle and Jimma in which contraceptive practice was found to be strongly associated with spousal discussion about FP, women’s perception of husband’s approval with contraceptive practices [18,22,23,27,28].

Even though this study is one among few studies conducted to assess the demand for LAPMs and associated factors, Lack of literatures which are specifically conducted on demand for long acting and permanent methods, being conducted only in the town and cross sectional study were some of its limitations.

## Conclusions

In conclusion, the total demand and overall practice of LAPMs in the town was higher when compared to the findings of other studies conducted in the country. Being in older age, having no desire for more child, desire to have a child after 2 years, having ever born children three or more, not ever heard of modern family planning, not ever using of modern FP, having no spousal discussion in the past six month about contraception, having no spousal discussion about which method to use and having perception of their husbands’ approval of using permanent methods after completing desired family size were significantly associated with having higher demand for LAPMs. Thus, the federal ministry of health and regional health bureau in combination with NGOs working on family planning have to work hard to increase accessibility and availability of LAPMs, because almost half of respondents had intention to use in the future and more than half had unsatisfied demand for LAPMs in the study area.

## Competing interests

The authors declared that they have no competing interests.

## Authors’ contributions

GA, wrote the proposal, participated in data collection, analyzed the data and drafted the paper. TA and TKB approved the proposal with some revisions, participated in data analysis and revised subsequent drafts of the paper. All authors read and approved the final manuscript.

## Pre-publication history

The pre-publication history for this paper can be accessed here:

http://www.biomedcentral.com/1472-6874/14/46/prepub

## References

[B1] Population Reference Bureau World Population Data Sheet 2012 Available at: http://www.prb.org/pdf12/2012-population-data-sheet_eng.pdf. Accessed on November 22, 2012

[B2] Summary and statistical report of 2007 population and housing census. Federal democratic republic of Ethiopia population census commission 2008 Addis Ababa, Ethiopia and Calverton Maryland, USA: Central Statistical Agency, United Nations Population Fund (UNFPA)

[B3] Ethiopian Demographic and Health Survey 2011 2011 Addis Ababa, Ethiopia: Central Statistical Agency and ICF International Accessed on December 11, 2011

[B4] Okonofua E Maternal mortality prevention in Africa needs to focus on Access and quality of care Afr J Reprod Health 2008 12 3 9 11 19435009

[B5] World Health Organization Maternal Mortality in 2005 Estimates developed by WHO, UNICEF, UNFPA, and World Bank 2007 4 19 Available at: http://www.whqlibdoc.who.int/publications/2007/9789241596213_eng.pdf. Accessed on December 12, 2011

[B6] Singh S Fetters T Gebreselassie H Abdella A Gebrehiwot Y Kumbi S Audam S The Estimated Incidence of Induced Abortion In Ethiopia Guttmacher Inst 2010 36 1 16 25 10.1363/ipsrh.36.016.10 20403802

[B7] Tsui A McDonald-Mosley R Burke A Family planning and burden of unintended pregnancyepirevoxfordjournalsorg 2010 32 1 152 174 10.1093/epirev/mxq012 PMC3115338 20570955

[B8] Pile M Ndede F Ndong I Jacobstein R Johre N Investing in the future the case for Long-acting and permanent contraception in Sub-Saharan Africa 2007 Arusha, Tanzania: AQUIRE project http://www.engenderhealth.org/files/pubs/acquire-digital-archive/2.0_invest_in_fp_and_lapms/2.2_resources/2.2.2_working_papers/investing_in_the_future.pdf. Accessed on December 12, 2011

[B9] World Health Organization Family planning a global hand book for providers 2011 updates 2011 3 WHO Available at: http://whqlibdoc.who.int/publications/2011/9780978856373_eng.pdf. Accessed on December 12, 2011

[B10] Say L Chou D Mommaerts M Haviland L Accelerating universal access to reproductive health in Ethiopia 2011 World Health Organization (WHO) RHR 11 19 Available at: http://www.who.int/reproductivehealth/publications/monitoring/rhr_hrp_11_19. Accessed on December 12, 2011

[B11] Family Health International Addressing Unmet Need for Family Planning in Africa 2007 New York, USA Available at: http://www.fhi.org/NR/rdonlyres/ejs4cfxbb2weisu5iqkq2om6ysamrht7mpvremntmhnong6onjlq6dwnwfuq5qpklqn44ms3dauxpd/LAPMbriefsall1.pdf. Accessed on December 12, 2011

[B12] USAID Long acting and permanent methods of contraception: Meeting clients’ needshttp://www.usaid.gov/our_work/global_health/pop/techareas/repositioning/briefs/lap_methods.pdf. Accessed on: December 12, 2011

[B13] World Health Organization Medical eligibiliity criteria for contraceptive use. Family planning cornerstone 2009 4 WHO 23741782

[B14] The Free Encyclopaedia Debre Markos town socio economic profile 2011 Accessible at http://en.wikipedia.org/wiki/Debre_Marqos. Accessed on: December 2, 2011

[B15] Mathe K Kasonia K Maliro K Barriers to adoption of family planning among women in Eastern Democratic Republic of Congo Afr J Reprod Health 2011 15 1 69 77 21987940

[B16] Ndaruhuye DM Broekhuis A Hooimeijer P Demand and Unmet Need for Means of Family Limitation in Rwanda Int Perspect Sex Reprod Health 2009 35 3 122 130 10.1363/3512209 19805017

[B17] Hailemariam A Haddis F Factors affecting unmet need for family planning in Southern Nations, Nationalities and Peoples region, Ethiopia Ethiop J Health Sci 2011 21 2 77 89 22434988 10.4314/ejhs.v21i2.69048 PMC3275860

[B18] Takele A Degu G Yitayal M Demand for long acting and permanent methods of contraceptives and factors for non-use among married women of Goba Town, Bale Zone, South East Ethiopia Reprod Health 2012 9 26 Epub 2012/10/30 10.1186/1742-4755-9-26 23102166 PMC3538527

[B19] Haile A Fantahun M Demand for long acting and permanent contraceptive methods and associated factors among family planning service users, Batu Jira town, Central Ethiopia Ethiop Med J 2012 50 1 31 42 22519160

[B20] Dibaba Y Factors influencing women’s intention to limit child bearing in Oromia, Ethiopia Ethiop J Health Dev 2009 23 1 28 33

[B21] Asnake M Walie L Melkamu Y Improving the range of contraceptive choices in rural Ethiopia Ethiop J Health Dev 2006 20 2 74 78

[B22] Agha S Intentions to use contraceptives in Pakistan: implications for behaviour change campaigns BMC Public Health 2010 10 450 463 10.1186/1471-2458-10-450 20673374 PMC2920282

[B23] Alemayehu M Belachew T Tilahun T Factors associated with utilization of long acting and permanent contraceptive methods among married women of reproductive age in Mekelle town, Tigray region, north Ethiopia BMC Pregnancy Childbirth 2012 12 6 Epub 2012/01/28 10.1186/1471-2393-12-6 22280163 PMC3297532

[B24] Hong R Montana L Mishra V Family planning services quality as a determinant of use of IUD in Egypt BMC Health Serv Res 2006 6 79 10.1186/1472-6963-6-79 16792810 PMC1553443

[B25] Khan S Bradley K Fishel J Mishra V Unmet Need and the Demand for Family Planning in Uganda: Further Analysis of the Uganda Demographic and Health Surveys, 1995–2006 2008 Calverton, Maryland, USA: Macro International Inc Available at: http://www.measuredhs.com

[B26] Ko IS You MA Kim ES Lee TW Kim S KIM YM Nam JJ Lee HK Family planning practice and related factors of married women in Ethiopia Int Nurs Rev 2010 57 377 382 10.1111/j.1466-7657.2010.00805.x 20796069

[B27] Beekle A McCabe C Awareness and determinants of family planning practice in Jimma, Ethiopia Int Nurs Rev 2006 53 269 276 10.1111/j.1466-7657.2006.00492.x 17083415

[B28] Haile A Enqueselassie F Influence of women’s autonomy on couple’s contraception use in Jimma town, Ethiopia Ethiop J Health Dev 2006 20 3 145 151

